# Double stranded DNA breaks and genome editing trigger loss of ribosomal protein RPS27A

**DOI:** 10.1111/febs.16321

**Published:** 2022-01-10

**Authors:** Celeste Riepe, Elena Zelin, Phillip A. Frankino, Zuriah A. Meacham, Samantha G. Fernandez, Nicholas T. Ingolia, Jacob E. Corn

**Affiliations:** ^1^ Department of Molecular and Cell Biology University of California, Berkeley CA USA; ^2^ Innovative Genomics Institute University of California, Berkeley CA USA; ^3^ Present address: Department of Biology ETH Zürich Switzerland

**Keywords:** DNA damage response, genome editing, ribosomes, translation

## Abstract

DNA damage activates a robust transcriptional stress response, but much less is known about how DNA damage impacts translation. The advent of genome editing with Cas9 has intensified interest in understanding cellular responses to DNA damage. Here, we find that DNA double‐strand breaks (DSBs), including those induced by Cas9, trigger the loss of ribosomal protein RPS27A from ribosomes via p53‐independent proteasomal degradation. Comparisons of Cas9 and dCas9 ribosome profiling and mRNA‐seq experiments reveal a global translational response to DSBs that precedes changes in transcript abundance. Our results demonstrate that even a single DSB can lead to altered translational output and ribosome remodeling, suggesting caution in interpreting cellular phenotypes measured immediately after genome editing.

## Graphical abstract

DNA damage activates a robust transcriptional stress response; however, how DNA damage impacts translation remains understudied. The emergence of Cas9‐based genome editing has intensified interest in elucidating cellular responses to DNA damage. Here, Nicholas Ingolia, Jacob Corn and colleagues describe how DNA double‐strand breaks (DSBs), including Cas9‐induced DSBs, trigger loss of ribosomal protein RPS27A from ribosomes. Comparison between Cas9 and dCas9 ribosome profiling and mRNA‐seq experiments reveal a translational response to DSBs that precedes transcriptional changes. The authors show that Cas9‐associated DSBs can cause altered translation and ribosome remodelling, suggesting caution in interpreting cellular phenotypes measured immediately after genome editing.

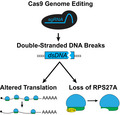

**Abbreviations**
4E‐BP: 4E‐binding protein
CML: chronic myeloid leukemia
DSBs: double‐stranded DNA breaks
eIF2α: eukaryotic initiation factor 2α
HDR: homology directed repair
ISR: integrated stress response
RNP: ribonucleoprotein
sgRNA: single guide RNA
UV: ultraviolet

## Introduction

Unrepaired DNA damage can lead to deleterious germline mutations and contributes to somatic cancer initiation and progression. Cells thus have evolved many responses to protect their genomes from a wide range of chemical and environmental insults. DNA double‐strand breaks (DSBs) pose a particularly acute danger, as they may cause the wholesale loss of genetic information and must be resolved through complex repair processes. In humans, cells with DSBs arrest until repair is completed and undergo programmed cell death if repair is unsuccessful.

Double‐strand breaks provoke a distinctive transcriptional response. Activation of the transcription factor p53 is a hallmark of the DSB response, leading to transcriptional reprogramming, cell cycle arrest, or in cases of severe damage, apoptosis [1]. Deficiency in p53 signaling is also pivotal to the progression of many cancers, allowing neoplasms to accumulate DNA damage that leads to mutations and rapid tumor evolution. In addition to its critical role in maintaining genomic integrity, the cellular response to DSBs plays an integral role in the most widely used genome‐editing methods. In these approaches, a programmable nuclease such as Cas9 introduces a targeted DSB within a genome, which the cell repairs through error‐prone non‐homologous end joining or through templated homology directed repair (HDR). In addition to activating these repair pathways, genome editing induces DNA damage response signals. HDR from even a single Cas9‐mediated DSB can induce low levels of p53 signaling with negative consequences for cell fitness and genome‐editing outcomes [2, 3].

Although DSBs are known to initiate changes in transcript abundance, less is understood about how DNA damage response impacts translation. Previous studies have demonstrated that translational output is reduced after different types of DNA damage, including the formation of thymidine dimers after ultraviolet (UV) exposure and the generation of multiple non‐specific DSBs by treatment with the anti‐cancer drug doxorubicin [4, 5, 6]. Both UV exposure and doxorubicin treatment have been shown to promote translation inhibition through the phosphorylation of eukaryotic initiation factor 2α (eIF2α) [5, 6, 7], which prevents eIF2 from recruiting the initiator methionine tRNA to the mRNA [8, 9]. Ionizing radiation has been shown to promote both the phosphorylation of eIF2α and the hypo‐phosphorylation of 4E‐binding protein (4E‐BP), which suppresses cap‐dependent translation by occluding the cap‐binding protein eIF4E [10, 11, 12]. UV exposure and ionizing radiation also alter translation of individual mRNA transcripts, including DNA damage response genes [13, 14]. Doxorubicin leads to extensive ribosomal ubiquitination, suggesting that ribosomes are post‐translationally modified after double‐stranded DNA damage [4]. However, it remains unknown if the relatively low levels of double‐stranded DNA damage incurred during Cas9 genome editing leads to reduced translational output, differential translation, or ribosome remodeling.

Here, we demonstrate that cells temporarily deplete a core ribosomal protein, RPS27A, in response to Cas9‐mediated DSBs. RPS27A is regulated post‐transcriptionally and in a p53‐independent manner, and its depletion persists days after the initial genomic lesion with Cas9. Ribosome profiling and RNA‐seq data from Cas9‐edited cells suggest that cells mount a translation response to DSBs that precedes changes in transcript abundance. Our data demonstrate that Cas9‐genome editing leads to changes in translation and ribosome composition that occur days after the initial DNA lesions.

## Results

### Core ribosomal protein RPS27A is lost from ribosomes after DSBs

**Fig. 1:**
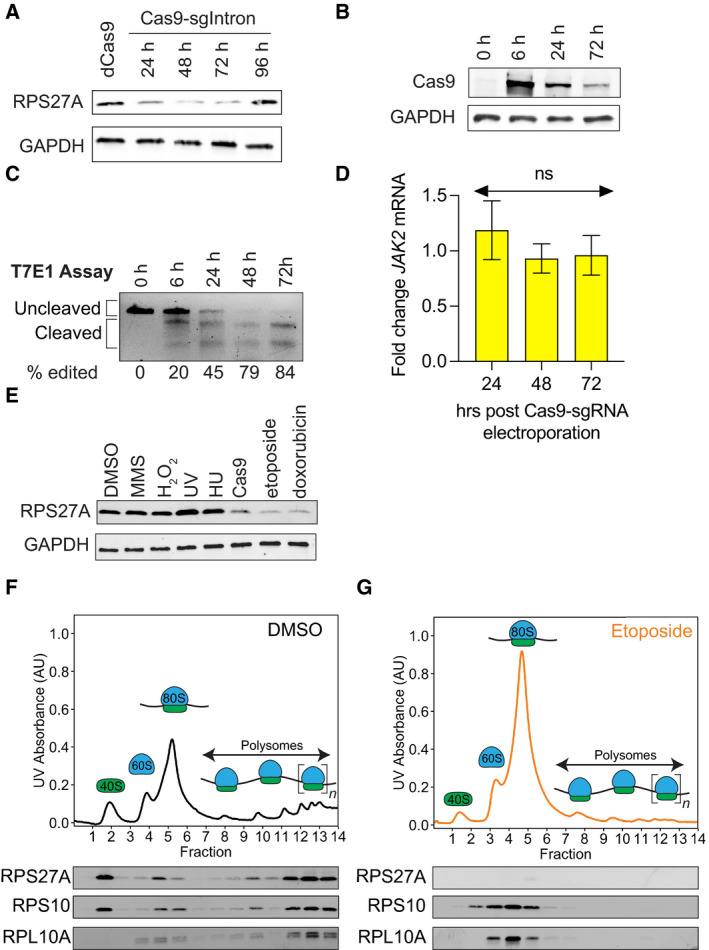
Core ribosomal protein RPS27A is lost from ribosomes after DSBs. (A) Western blots reveal that RPS27A is depleted in parental HEK cells after electroporation with Cas9‐sgIntron (sgJAK2) RNPs. HEK cells harvested 72 h post dCas9‐sgIntron electroporation served as the negative control. (B) Western blots depict loss of Cas9 protein after Cas9‐sgIntron electroporation. (C) T7 endonuclease 1 (T7E1) assay of *JAK2* editing after Cas9‐sgIntron electroporation. Band intensities were calculated using imagej, and percent edited was calculated as 100% × (1 − (1 − fraction cleaved)^1/2^), where fraction cleaved = (sum of cleavage product intensities)/(sum of uncleaved and cleaved product intensities). (D) Genome editing does not affect *JAK2* mRNA abundance. Fold changes were calculated using the$2^{‐\Delta \Delta C_{t}}$ method with Cas9 without sgIntron (apo Cas9) as the control and *GAPDH* as the reference gene (*n* = 3, error bars = standard deviation, *P* > 0.05, one‐way ANOVA). (E) Western blots show that RPS27A is depleted after DNA DSBs but not after other forms of DNA damage. MMS: methyl methanesulfonate, 0.03%, 1 h. Cas9: Cas9*‐*sgIntron electroporation, 72 h recovery. H_2_O_2_: 500 μm hydrogen peroxide, 1 h. UV: UV irradiation, 20 J·m^−2^, 6 h recovery. HU: hydroxyurea, 10 mm, 16 h. Etoposide: 5 μm, 16 h. Doxorubicin: 10 μm, 16 h. (F, G) Polysome profiles and western blots of polysome profiling fractions from HEK cells treated with (F) DMSO or (G) 5 μm etoposide for 16 h reveal that RPS27A is lost from ribosomes after DSBs. UV absorbance = UV absorbance at 254 nm.

While investigating changes in ubiquitin gene expression after DNA damage, we serendipitously observed that RPS27A (eS31) is downregulated after Cas9‐single guide RNA (sgRNA) ribonucleoprotein (RNP) electroporation (Fig. 1A). This downregulation was apparent as late as 48–72 h after electroporation, even though at this point, the cells had turned over most of the Cas9 RNPs (Fig. 1B) and formation of indels was complete (Fig. 1C). RPS27A levels recovered within 96 h after electroporation (Fig. 1A).

Downregulation of RPS27A depended on the DNA DSB, as catalytically inactive dCas9 did not provoke a similar response (Fig. 1A). The guide RNA used in this experiment targeted a non‐coding region of the *JAK2* gene (sgIntron), and *JAK2* levels remain unchanged after Cas9 electroporation (Fig. 1D). Our data therefore suggest that the loss of RPS27A was due to the break itself and not disruption of *JAK2*. This days‐long response was striking, as Cas9‐mediated genome editing is often assumed to be relatively benign beyond the effects of the genomic sequence change itself.

We next asked whether RPS27A depletion was a specific response to DSBs versus other genomic lesions. Loss of RPS27A did not occur after non‐DSB DNA damage such as alkylation (methyl methanesulfonate), oxidative damage (hydrogen peroxide), thymine dimers (UV radiation), or replication fork stalling (hydroxyurea) (Fig. 1E). By contrast, both single, targeted DSBs caused by Cas9 RNP electroporation and multiple, unspecific DSBs induced by the topoisomerase II inhibitors etoposide or doxorubicin reduced RPS27A levels. Therefore, the loss of RPS27A after genome editing is caused by multiple DSB‐inducing agents and is specific to DSBs.

As RPS27A is a core component of the ribosome, we wondered whether intact ribosomes lacked this core component or if the reduction in its level after DSBs reflected changes in the pool of free ribosomal subunits. We used western blotting of sucrose density gradient fractions to measure the abundance of different ribosomal proteins in small (40S) and large (60S) ribosome subunits, 80S monosomes, and polysomes from cells treated with DMSO or etoposide (Fig. 1F,G). Etoposide caused an accumulation of 80S monosomes and a reduction of actively translating polysomes. RPS27A was absent from 80S monosomes and other ribosomal subunits after etoposide treatment while the control ribosomal proteins RPS10 (eS10) and RPL10A (uL1) remained in all ribosomal species. The lack of RPS27A in 80S monosomes and polysomes suggests that it is absent from actively translating ribosomes, but we cannot rule out that monosomes are not translationally competent after DSBs and that actively translating ribosomes require RPS27A.

### RPS27A is proteasomally degraded in response to DSBs

**Fig. 2:**
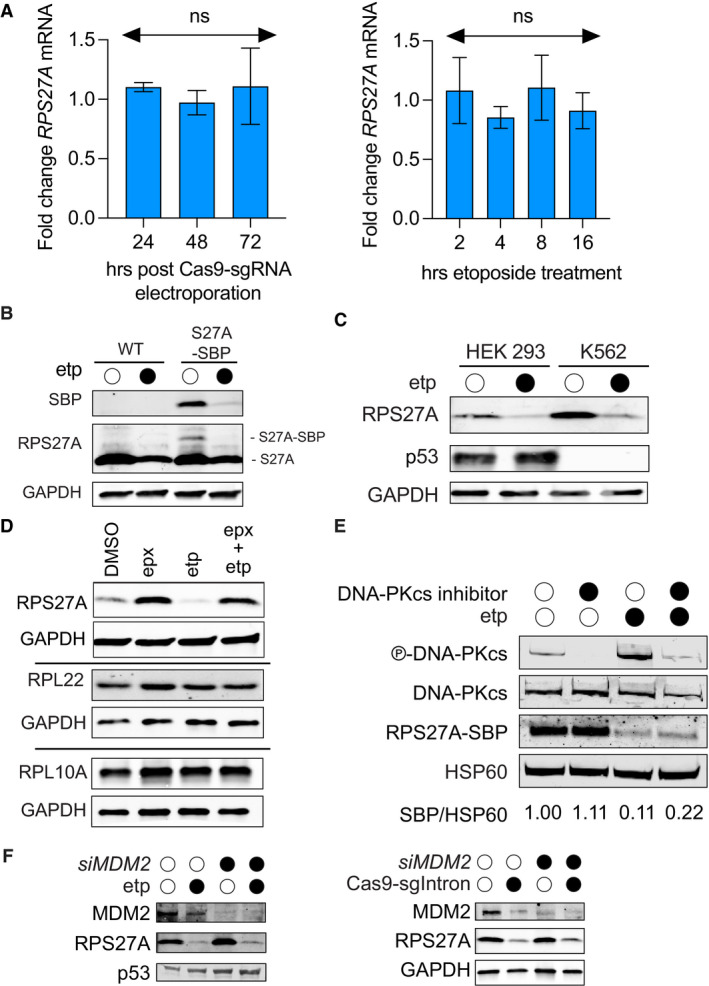
RPS27A is proteasomally degraded in response to dsDNA damage. (A) *RPS27A* transcript levels do not change after Cas9‐sgIntron electroporation or 5 μm etoposide treatment. mRNA fold changes were calculated using the $2^{‐\Delta \Delta C_{t}}$ method with Cas9 without sgIntron (apo Cas9) or DMSO as the controls and *GAPDH* as the reference gene (*n* = 3, error bars = standard deviation, *P* > 0.05, one‐way ANOVA). (B) Western blotting of RPS27A and SBP in HEK Flp‐In cell lines carrying a *pCMV‐RPS27A‐SBP* transgene. Cells were treated with 5 μm etoposide for 16 h. (C) Western blots confirm the p53 null status of K562 cells and demonstrate that loss of RPS27A is p53‐independent. (D) Proteasome inhibition with epoxomicin rescues RPS27A upon dsDNA damage. Cells were treated with 50 nm epoxomicin for 1 h before cells were treated with 5 μm etoposide and/or 50 μm epoxomicin for 16 h. (E) Chemical inhibition of DNA‐PKcs mitigates the loss of RPS27A‐SBP after etoposide treatment. HEK Flp‐In RPS27A‐SBP cells were pretreated with 2 μm NU7441 for 1 h before co‐administration with 5 μm etoposide for 16 h. phospho‐DNA‐PKcs (S2056) served as the positive control for DNA‐PKcs inhibition. Fold changes in RPS27A‐SBP levels represent the normalized ratio of SBP to HSP60 signal measured with the LI‐COR Image Studio software. (F) Western blotting demonstrates RPS27A depletion is insensitive to *MDM2* knock‐down after etoposide treatment or Cas9‐sgIntron electroporation. DMSO served as the negative control for etoposide, non‐targeting siRNA served as the negative control for *siMDM2*, and Cas9 without a guide served as the negative control for Cas9‐sgIntron.

We found that loss of RPS27A was post‐transcriptional, as mRNA levels of *RPS27A* did not change in response to DSBs induced by either etoposide or Cas9 (Fig. 2A). Moreover, a constitutively expressed transgene of RPS27A fused to an SBP tag also decreased after etoposide treatment (Fig. 2B). RPS27A loss was independent of the p53‐mediated transcriptional response since the level of RPS27A was reduced after DSBs in both p53‐positive (HEK293) and p53‐negative (K562) cell lines (Fig. 2C).

We therefore asked if proteasomal degradation could explain the loss of RPS27A. Indeed, proteasome inhibition by epoxomicin treatment rescued the loss of RPS27A after DNA damage (Fig. 2D). Epoxomicin treatment also increased basal levels of RPS27A, suggesting a substantial level of constitutive RPS27A degradation. Levels of other ribosomal proteins, including RPL22 and RPL10A, were unchanged by etoposide or epoxomicin treatment, suggesting that this response is specific for RPS27A and does not globally affect ribosomal proteins. We also sought to identify the DNA damage signal that promotes RPS27A degradation, and we found that chemically inhibiting DNA damage response kinase, DNA‐PKcs, led to a two‐fold rescue of RPS27A‐SBP after etoposide treatment (Fig. 2E).

Proteasomal degradation is initiated by E3 ubiquitin ligases, which play a prominent role in several aspects of DNA damage signaling. MDM2 is a DNA damage regulated ubiquitin ligase that targets p53 for degradation under normal growth conditions and has been reported to ubiquitinate RPS27A [15]. However, we found that siRNA knockdown of MDM2 had no effect on the depletion of RPS27A caused by etoposide or Cas9 (Fig. 2F), further suggesting that loss of RSP27A is independent of the p53 pathway. Taken together, our data indicate that cells lose mature RPS27A through proteasome‐mediated degradation through a p53‐independent mechanism.

### Protein synthesis decreases after DSBs

**Fig. 3:**
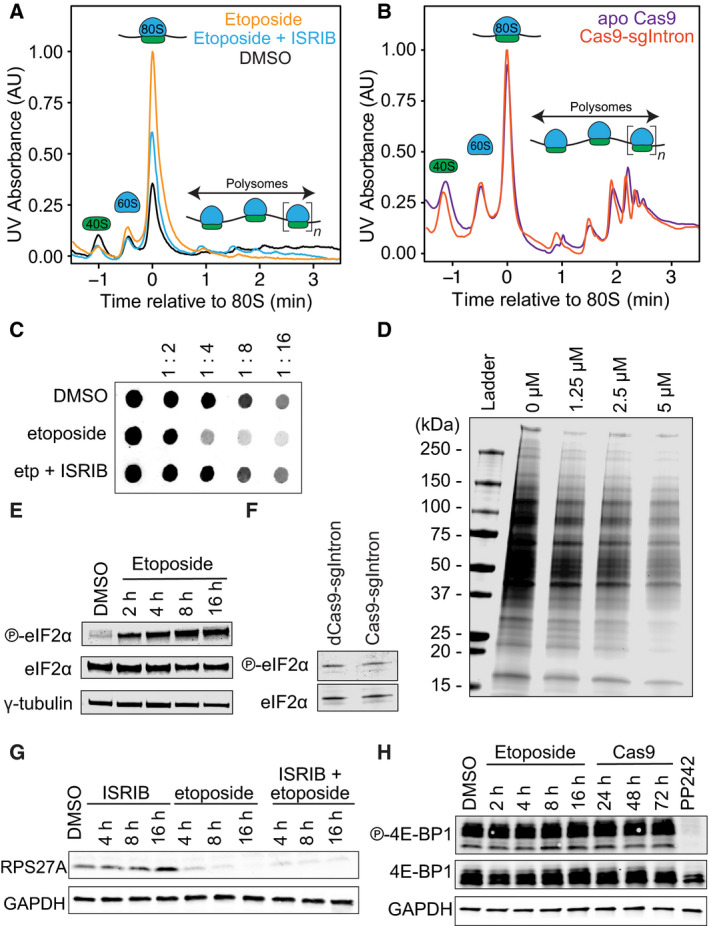
Protein synthesis decreases after DSBs. (A) Polysome profiles of HEK cells treated with 5 μm etoposide or 5 μm etoposide and 200 nm ISRIB for 16 h. (B) Polysome profiles of HEK cells 72 h after electroporation with Cas9‐sgIntron or Cas9 without guide (apo Cas9). (C) l‐azidohomoalanine (AHA) bulk translation assay demonstrates that DSBs reduce protein synthesis. HEK cells were lysed after 16 h after treatment with 5 μm etoposide and 200 nm ISRIB. Two hours before lysis, growth media was replaced with methionine‐free media containing a methionine mimic, l‐azidohomoalanine (l‐AHA). Lysates were normalized by protein content, labeled with IRDye 800CW‐DBCO, blotted on a nitrocellulose membrane, and imaged with a LI‐COR Odyssey CLx Imager. (D) l‐ AHA bulk translation assay demonstrates that the degree of DNA damage influences the magnitude of translational repression. HEK cells were dosed with different concentrations of etoposide for 16 h, and the AHA assay was performed as detailed in (C) with the exception that samples were run on an SDS/PAGE gel. (E) eIF2α (S51) phosphorylation increases in HEK cells treated with 5 μm etoposide for 16 h. (F) eIF2α (S51) phosphorylation does not increase in HEK cells 72 h post Cas9‐sgIntron electroporation. (G) Western blotting indicates that co‐administration of 200 nm ISRIB with 5 μm etoposide does not rescueRPS27A 16 h post drug treatment. (H) 4E‐BP1 (T37/47) phosphorylation does not change after dsDNA damage. Cells were either treated with 5 μm etoposide or electroporated with Cas9‐sgIntron. Treatment with 2.5 μm PP242 for 30 min served as a positive control for 4E‐BP1 hypo‐phosphorylation.

Given that the core ribosomal subunit RPS27A is degraded after etoposide treatment and Cas9‐mediated genome editing, we asked whether bulk translation changes after DSBs. We performed polysome profiling to measure the translational status of HEK293 cells after non‐specific DSBs or genome editing. Etoposide treatment caused an accumulation of 80S monosomes and a reduction of actively translating polysomes (Fig. 3A). Etoposide‐treated cells also exhibited an imbalance between small (40S) and large (60S) ribosome subunits relative to the ratio seen in DMSO‐treated samples (40S : 60S peak height ratio of 2 : 7 for etoposide versus 1 : 1 for DMSO), suggesting a deficiency in 40S subunits. DSBs induced by Cas9 editing led to a more subtle change in the polysome profile, with a modest increase in 80S monosomes, decrease in 40S subunit, and shift from heavy to light polysomes (Fig. 3B).

**Fig. 4:**
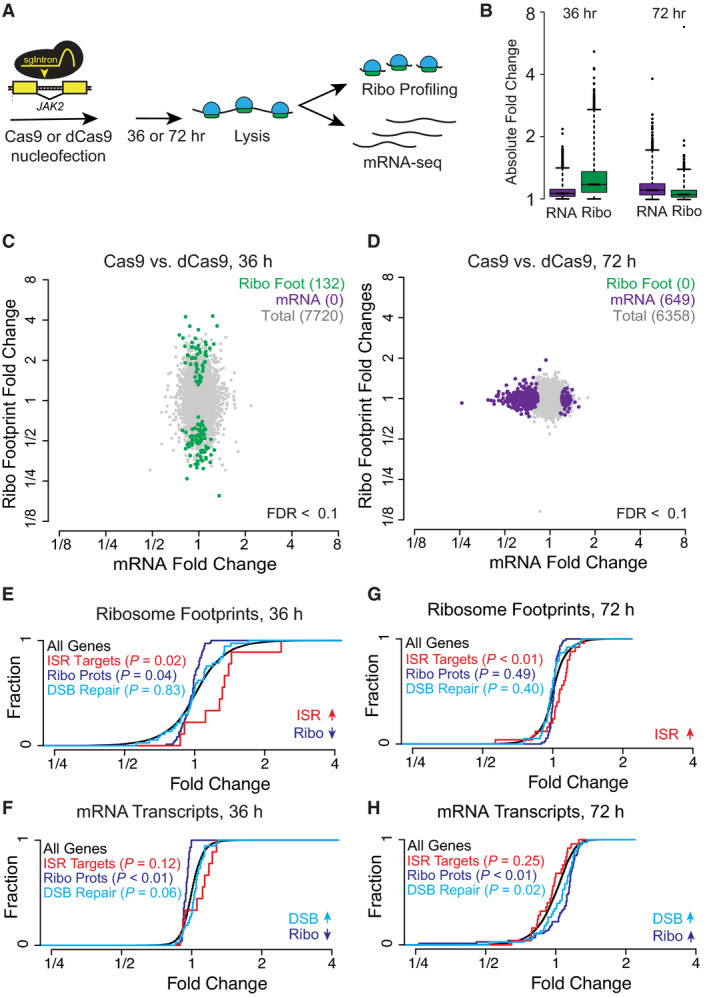
Genome editing initiates a translational response that precedes changes in transcript abundance. (A) Experimental design for ribosome profiling and RNA‐seq experiments. HEK cells were electroporated with Cas9‐sgIntron or dCas9‐sgIntron and harvested after 36 or 72 h. Lysates were divided between ribosome profiling and RNA‐seq experiments. (B) Distribution of absolute fold changes on a logarithmic scale for genes identified in RNA‐seq and ribosome profiling experiments at 36 and 72 h post editing. Whiskers denote values 1.5 × (the interquartile range). (C, D) Changes in ribosome footprint versus mRNA abundance at 36 h (C) and 72 h (D) after Cas9 or dCas9 electroporation. Green = genes with significant changes in ribosome footprints. Purple = genes with significant changes in mRNA transcripts (Wald test, FDR < 0.1). (E–H) Cumulative distribution function (CDF) plots for ribosomal protein genes (Ribo), ISR targets, and DSB repair genes observed in the ribosome profiling (E, G) and mRNA‐seq (F, H) experiments 36 h (E, F) or 72 h (G, H) after Cas9‐sgIntron electroporation. *P*‐values were calculated using the Mann–Whitney–Wilcoxon rank sum test. See Table S2 for target set gene lists.

Because an increase in 80S monosomes is a hallmark of reduced protein synthesis, we asked how bulk translation changes after DSBs. We tracked nascent protein synthesis by culturing DNA‐damaged cells in media containing l‐azidohomoalanine (AHA), a methionine mimic that can be labeled with alkyne‐conjugated probes [16]. Induction of high levels of non‐specific DSBs with etoposide led to a marked reduction in translation (Fig. 3C). This reduction in translation was dependent on the concentration of etoposide, suggesting that the degree of DNA damage influences the magnitude of translational repression (Fig. 3D).

We next sought to identify the mechanism by which cells inhibit translation after DSBs. We asked if dsDNA‐damaged cells regulate translation through either of two canonical mechanisms: the phosphorylation of eIF2α or the de‐phosphorylation of 4E‐BP. Phosphorylation of eIF2α prevents eIF2 from recruiting the initiator methionine tRNA to the mRNA [8, 9] while 4E‐BP suppresses cap‐dependent translation by preventing eIF4E from interacting with other components of the eIF4F complex [10, 11, 12]. We found that etoposide induced phosphorylation of eIF2α (Fig. 3E), but Cas9‐mediated DSBs did not induce a visible response (Fig. 3F). We confirmed that phosphorylation of eIF2α causes translation repression after etoposide treatment by demonstrating that ISRIB, a small molecule that mitigates the downstream effects of eIF2α phosphorylation [17], rescues the etoposide‐induced accumulation of 80S monosomes, depletion of polysomes, 40S : 60S imbalance, and reduction in protein synthesis (Fig. 3A,C). However, ISRIB treatment did not rescue RPS27A levels after etoposide treatment, suggesting that the degradation of RPS27A is not dependent on the downstream stress responses activated after eIF2α phosphorylation (Fig. 3G). Neither Cas9 editing or etoposide reduced the phosphorylation of inhibitory 4E‐BP (Fig. 3H), suggesting that the mechanism of translational repression is independent of this pathway.

### Genome editing initiates a translational response that precedes changes in transcript abundance

Given that Cas9‐induced DSBs trigger ribosome remodeling, we wanted to determine whether genome editing impacts translation of individual genes. We carried out matched ribosome profiling and mRNA sequencing at 36 and 72 h after electroporation of HEK293 cells with Cas9‐sgIntron or catalytically dead dCas9‐sgIntron (Fig. 4A). At 36 h after Cas9 electroporation, we found 132 genes that exhibit changes in ribosome footprint abundance while no genes changed in transcript abundance (Wald test, FDR corrected *P*‐value < 0.1, Fig. 4B,C, Table S1). On the other hand, at 72 h after electroporation there were changes in mRNA transcript levels but no statistically significant changes in footprint abundance (Fig. 4B,D, Table S1). Translational efficiency, the ratio of ribosome footprints to mRNA transcripts, reflected these differences in transcription and translation. Changes in translational efficiency were driven by translation at 36 h and by mRNA abundance at 72 h (Fig. S1A,B). Thus, genome editing activates a translational program that precedes changes in transcript abundance.

We next asked if there were coordinated shifts in gene expression of DNA repair factors after Cas9 electroporation. We found that DSB repair genes showed no significant change in ribosome footprints or translation efficiency at 36 h (Fig. 4E and Fig. S1C) but a small decrease in translation efficiency at 72 h that was driven by increased transcript abundance without a concomitant change in ribosome footprints (Fig. 4H and Fig. S1C). This rebalancing could reflect cells tuning down production of DSB repair proteins as cells return to homeostasis after editing.

We also observed a significant but modest activation of the integrated stress response (ISR) after Cas9 electroporation. The ISR is a homeostatic gene expression program that is translationally activated in response to a diverse set of stressors including amino acid starvation, viral infection, hypoxia, and protein misfolding [18, 19]. ISR activation generally occurs downstream of eIF2α phosphorylation, but we could not detect an increase in phospho‐eIF2α after Cas9 electroporation (Fig. 3F). Etoposide treatment, on the other hand, did induce eIF2α phosphorylation. Therefore, it seems possible that the phosphorylation occurring after Cas9 electroporation is too weak or heterogeneous in time to observe by immunoblotting. We defined ISR targets (Table S2) as the subset of genes translationally regulated after cells were treated with tunicamycin, an inhibitor of N‐linked glycosylation that upregulates eIF2α phosphorylation [20], and sensitive to the co‐administration of ISRIB, which mitigates the downstream effects of eIF2α phosphorylation. At both 36 and 72 h after Cas9 electroporation, we found that ISR targets exhibited increases in translation but not transcription (*P* < 0.05, Mann–Whitney–Wilcoxon test, Fig. 4E–H), supporting a model in which Cas9 genome editing can activate the translation of ISR targets through the phosphorylation of eIF2α.

Given that reduced translation of ribosome protein genes has been observed after ISR has been activated [20], we asked if similar reductions in ribosome protein expression arise after Cas9‐mediated DSBs. We found decreased footprints and mRNA abundance for several ribosomal protein transcripts 36 h after Cas9 editing (*P* < 0.05, Mann–Whitney–Wilcoxon test, Fig. 4E–F), but ribosome protein transcript levels increased 72 h after a Cas9‐mediated DSB, suggesting that the cell may reset ribosome protein levels through increased transcription (Fig. 4G,H).

## Discussion

DNA damage poses a serious threat to genome integrity. Consequently, cells have an array of responses dedicated to mitigating this threat. The transcriptional response to DNA double strand breaks, mediated by p53, is a well‐studied example that has recently been appreciated to play a role during CRISPR‐Cas genome editing [2, 3]. Here we report that both nonspecific dsDNA damage and Cas9 genome editing lead to the loss of the core ribosomal protein RPS27A. These data highlight how Cas9 genome editing can cause phenotypes independent of the intended edit.

### Ribosomes lose core ribosomal protein RPS27A after DSBs

Our observation that ribosomes lack RPS27A after dsDNA damage is one of the few known instances where ribosome composition is modulated in response to a specific biological stimulus [21, 22]. While differential expression of ribosomal proteins between tissue types and subpopulations of ribosomes within a cell are emerging themes in ribosome biology, there have been few reports of human cells altering ribosome composition in response to the cellular environment. While loss of RPS27A may alter ribosome function in a way that is difficult to detect in our ribosome profiling analysis, we cannot rule out that ribosomes lacking RPS27A carry out specialized translation.

Loss of RPS27A could also prevent 40S maturation. It was recently reported that mono‐ubiquitination of RPS27A only occurs in actively translating cells and is important for 40S maturation [23]. The complete loss of RPS27A after DSBs could therefore prevent downstream recognition of mono‐ubiquitinated RPS27A, halting maturation of 40S subunits in the cell and leading to the reduction in global translational output and decrease in free 40S we observe. Further studies may concretely link molecular regulation of RPS27A abundance and modification to the maintenance of global translation.

Alternatively, we cannot rule out that the depletion of RPS27A may serve to regulate an extra‐ribosomal function of the protein. RPS27A was previously reported to bind and inhibit the E3 ligase MDM2 [15], thereby promoting p53 expression in the cell. However, we found no evidence of reciprocal regulation since knockdown of MDM2 did not affect the reduction of RPS27A in response to DSBs (Fig. 2F). RPS27A was also shown to be elevated in chronic myeloid leukemia (CML) and acute leukemia, and knockdown of RPS27A in CML K562 cell lines decreased cell proliferation, arrested cells at the S and G2/M phases, and promoted apoptosis [24]. Moreover, knockdown was shown to inactivate the Raf/MEK/ERK signaling pathway and upregulate the p21 tumor suppressor, which inhibits cell cycle progression. Therefore, depletion of RPS27A after DSBs may serve as a signal to promote cell cycle arrest.

### Genome editing leads to global changes in gene expression arising from the dsDNA damage response

There is growing appreciation that Cas9 genome editing can cause cellular effects beyond the intended edit that involve a muted version of the response to multiple, non‐specific DSBs. For example, embryonic stem cells are hyper‐sensitive to HDR from even a single DSB introduced by Cas9, and such DNA damage can induce a p53 response that compromises cell health [2, 3]. CRISPR‐Cas9 nuclease screening data have also shown that targeting high copy number or repetitive regions of a genome reduces cell fitness, consistent with a graded cell cycle arrest that could be caused by p53 signaling [25, 26, 27].

Much of the concern about the safety and efficacy of genome editing has focused on off‐target mutagenesis, but our findings highlight that activation of the endogenous DNA damage response during Cas9 genome editing can have an impact on a cell's translatome and transcriptome independent of the gene target. These cellular responses should be taken into account when it is impossible to isolate and expand a clonal cell line for long periods of time after genome editing. Therefore, inclusion of non‐coding editing controls such as Cas9‐sgIntron may be more appropriate negative controls for short‐term knockout experiments than catalytically inactive dCas9 RNPs or Cas9 electroporations without a guide RNA.

## Materials and methods

### Cell culture

HEK 293 cells (ATCC, Manassas, VA, USA) were cultured in DMEM, high glucose, GlutaMAX (Thermo Fisher Scientific, Waltham, MA, USA) with 10% FBS (VWR, Radnor, PA, USA), or 10% FBS 1 mM sodium pyruvate, and penicillin–streptomycin in a 37 °C incubator with 5.0% CO_2_ and 20% O_2_. K562 cells (ATCC) were cultured in RPMI, GlutaMAX (Thermo Fisher Scientific) with 10% FBS (VWR), 10% sodium pyruvate.

### Inducing DNA damage

For chemically inducing double‐stranded DNA damage, HEK cells were grown to 70% confluency then treated for 16 h with 5 μm etoposide (Millipore Sigma, St. Louis, MO, USA) or 10 μm doxorubicin (Millipore Sigma). For chemically inducing other forms of DNA damage, HEK cells were treated with 0.03% methyl methanesulfonate for 1 h, 500 μm hydrogen peroxide for 1 h, or 10 mm hydroxyurea for 16 h. To damage cells using UV light, cells were irradiated at 20 J·m^−2^ with a FB‐UVXL‐1000 UV Crosslinker (Thermo Fisher Scientific) and recovered for 6 or 24 h before lysis. Cells were treated with DMSO for 16 h as a negative control unless otherwise noted.

### Cas9 RNP electroporation

sgRNAs were *in vitro* transcribed as previously described [28, 29]. In brief, the sgRNA transcription template contained a T7 RNA pol promoter followed by target specific region and constant region (T7FwdVar for sgIntron, GGATCCTAATACGACTCACTATAGACCATCACCCTCGAGGTACGTTTTAGAGCTAGAA) along with a primer that is the reverse complement of the invariant region of T7FwdVar (T7RevLong, AAAAAAGCACCGACTCGGTGCCACTTTTTCAAGTTGATAACGGACTAGCCTTATTTTAACTTGCTATTTCTAGCTCTAAAAC) and amplification primers (T7FwdAmp, GGATCCTAATACGACTCACTATAG, and T7RevAmp, AAAAAAGCACCGACTCGG). Transcription templates for sgRNA synthesis were PCR amplified from the primer mix, and Phusion High Fidelity DNA polymerase was used for assembly (New England Biolabs, Ipswich, MA, USA). Assembled template was used without purification for *in vitro* transcription by T7 polymerase using the HiScribe T7 High Yield RNA Synthesis Kit (NEB). RNA was purified with the Qiagen RNeasy kit (Qiagen, Hilden, Germany), and Cas9 and dCas9 RNPs were prepared as detailed in Ref. [29]. IVT sgRNAs were used in all experiments except for the 36‐h ribosome profiling and the Cas9 eIF2α western blotting experiments, which used synthetic sgRNAs (Synthego, Menlo Park, CA, USA).

HEK cells were passaged 2 days before electroporation and trypsinized at 60–90% confluency. For RNP electroporations, either 100 pmol Cas9 and 120 pmol gRNA were added to 2.5 × 10^5^ cells in 20 μL SF Solution (Lonza, Basel, Switzerland) or 300 pmol Cas9 and 300 pmol gRNA were added to 1 × 10^6^ cells suspended in 100 μL SF Solution (Lonza). HEK cells were electroporated using program CM‐130 in the X Unit of a Lonza 4D‐Nucleofector (AAF‐1002X, AAF‐1002B) and prewarmed media was immediately added to the cuvettes to increase cell viability. K562 cells were electroporated with Cas9 RNPs as described for HEK cells using buffer SF and program FF‐120.

### Drug treatment of HEK cells

To prevent proteasomal degradation during DNA damage, cells were treated with 50 nm epoxomicin (Millipore Sigma) for 1 h before cells were treated with 5 μm etoposide and/or 50 μm epoxomicin for 16 h. Cells were pretreated with 2 μm NU7441 (Selleck Chemicals, Houston, TX, USA) for 1 h before the drug was co‐administered with 5 μm etoposide for 16 h to investigate the role of DNA‐PKcs signaling in RPS27A degradation. To rescue downstream effects of eIF2ɑ phosphorylation after DNA damage, 200 nM ISRIB (Millipore Sigma) was added at the same time as etoposide. Cells were treated with 2.5 μm PP242 (Millipore Sigma) for 30 min as a control for 4E‐BP1 hypo‐phosphorylation. DMSO served as a negative control unless otherwise noted.

### siRNA knockdowns

siRNA oligos were transiently transfected into cells using RNAiMAX (Invitrogen, Waltham, MA, USA) according to the manufacturer's instructions. For each well of a 12‐well plate, 120 pmol siRNA and 3.6 μL RNAiMAX were used. Cells were transfected with siRNAs 24 h prior to drug treatment or Cas9 electroporation. siRNAs for this study were Human ON‐TARGETplus SMARTpool *siMDM2* (Dharmacon, Lafayette, CO, USA) and ON‐TARGETplus Non‐Targeting siRNA Pool (Dharmacon).

### Western blotting

Lysates were prepared using one of the three methods. In the first method, cells were pelleted at 400 ***g*** for 5 min then washed twice with PBS before being lysed in RIPA buffer with 1× Halt Protease Inhibitor Cocktail or 1× Halt Protease and Phosphatase Inhibitor Cocktail (Thermo Fisher Scientific) with 150 U·mL^−1^ benzonase nuclease (Millipore Sigma) to digest DNA and RNA. Lysates were incubated for 30 min on ice, vortexed for 30 s, and spun at 18 000 ***g*** for 10 min at 4 °C. Lysates were normalized using BCA (Thermo Fisher Scientific) or Bradford assays (Proteomics Grade; VWR) before being boiled at 97 °C for 5 min with Laemmli buffer or Novex LDS Sample Buffer (Thermo Fisher Scientific). Samples were loaded onto NuPAGE 4–12% Bis‐Tris (Thermo Fisher Scientific) and run in MES running buffer for 200 V for 40 min.

In the second method, HEK cells were washed with DPBS then lysed with ice cold polysome buffer (20 mm Tris pH 7.4, 150 mm NaCl, 5 mm MgCl_2_, and 1 mm DTT) with 1% Triton X‐100, 25 U·mL^−1^ TURBO DNase (Thermo Fisher Scientific), protease inhibitor cocktail (P1860; Millipore Sigma), and 1× Halt Phosphatase Inhibitor Cocktail (Thermo Fisher Scientific). Lysates were incubated on ice for 10 min then spun at 20 000 ***g*** to remove cellular debris. Lysates were normalized using the Pierce 660 nm protein assay (Thermo Fisher Scientific). Samples were heated in 1× LDS loading buffer for 10 min at 70 °C and run on Bolt 4–12% Bis‐Tris (Thermo Fisher Scientific) gels with MES buffer according to the manufacturer's protocol.

In the third method, HEK cells were washed with DPBS then lysed in 140 mm KCl, 10 mm HEPES, 5 mm MgCl_2_, 1% Triton‐X, 1 mm TCEP with cOmplete Protease Inhibitor Cocktail (Millipore Sigma), Halt phosphatase inhibitor, and TURBO DNase. Samples were heated in Laemmli buffer for 5 min at 97 °C and run on 4–20% Mini‐PROTEAN TGX gels (Bio‐Rad, Hercules, CA, USA) in 1× Tris‐Glycine SDS buffer (Thermo Fisher Scientific) at 200 V for 40 min.

After SDS/PAGE, proteins were transferred onto nitrocellulose membranes using the Trans‐Blot Turbo Blotting System (Bio‐Rad) according to the manufacturer's protocol using the Standard SD, 1.5 mm High MW program. Membranes were blocked in 5% milk in TBST for 15 min, washed 3 × 5 min in TBST, and incubated with primary antibodies in 5% BSA in TBST overnight at 4 °C. Membranes were washed 3 × 5 min in TBST and incubated with either IRDye 800CW (LI‐COR, Lincoln, NE, USA), IRDye 680RD (LI‐COR), or HRP‐conjugated secondary antibodies in 5% milk for 40 min before 2 × 5 min washes with TBST and 1 × 5 min wash with PBS. Blots were imaged by a LI‐COR Odyssey CLx Imager or Pierce ECL reagents (Thermo Fisher Scientific) and X‐ray film.

### Antibodies

The following primary antibodies were used for this study (dilutions were 1 : 1000 unless otherwise noted): 4E‐BP1 Rabbit Polyclonal Ab (Cell Signaling Technology [CST], Danvers, MA, USA; Cat# 9452), Phospho‐4E‐BP1 (T37/46) Rabbit Monoclonal Ab, Clone 236B4 (CST; Cat# 2855), GAPDH Rabbit Monoclonal Ab, Clone 14C10 (CST; Cat# 2118), eIF2α Rabbit Polyclonal Ab (CST; Cat# 9722), eIF2α Mouse Polyclonal Ab (CST; Cat# 2103), Phospho‐eIF2α (S51) XP Rabbit Monoclonal Ab, Clone D9G8 (CST; Cat# 3398), γ‐Tubulin Rabbit Polyclonal Ab (Santa Cruz Biotechnology, Dallas, TX, USA; Cat# sc‐7396‐R), RPS27A Mouse Monoclonal Ab, Clone 3E2‐E6 (Abcam, Cambridge, UK; Cat# ab57646), Cas9 Mouse Monoclonal Ab, Clone 7A9‐3A3 (Active Motif, Carlsbad, CA, USA; Cat# 61578), RPS10 Rabbit Polyclonal Ab (Novus, Littleton, CO, USA; Cat# NBP1‐98599), RPL10A Rabbit Polyclonal Ab (Bethyl, Montgomery, TX, USA; Cat# A305‐062A), RPL22 Rabbit Polyclonal Ab (Abcam; Cat# ab77720), DNA‐PKcs Rabbit Polyclonal Ab (1 : 5000 dilution; CST, Cat# 4602), Phospho‐DNA‐PKcs (Ser2056) Rabbit Monoclonal Ab, EPR5670 (Abcam; Cat# ab124918), P53 Mouse Monoclonal Ab, Clone DO‐1 (1 : 500; Santa Cruz Biotechnology, Cat# sc‐126), SBP Tag Mouse Monoclonal Ab, Clone SB19‐C4 (Santa Cruz Biotechnology; Cat# sc‐101595), and MDM2 Mouse Monoclonal Ab, Clone SMP14 (Santa Cruz Biotechnology; Cat# sc‐965).

### T7 endonuclease 1 assay

Edited cells were gathered off of plates with a pipette, spun at 10 000 ***g*** for 1 min, washed once with PBS, and lysed in QuickExtract™ DNA Extraction Solution (Lucigen, Middleton, WI, USA). Lysates were incubated at 65 °C for 6 min and 98 °C for 2 min in a thermocycler. Edited regions of the *JAK2* gene were PCR amplified in 100 μL reactions with AmpliTaq Gold 360 Master Mix (Thermo Fisher Scientific) with forward primer CCTCAGAACGTTGATGGCAGTT and reverse primer CTCTATTGTTTGGGCATTGTAACC. PCR products were purified using MinElute PCR Purification Kit (Qiagen). PCR products were hybridized and digested with T7 endonuclease 1 (NEB) according to the NEB protocol for determining targeting efficiency. Digests were run on a 2% agarose gel, and relative intensities from DNA bands were quantified using imagej [30] with % edited = 100 × (1 − (1 − fraction cleaved)^1/2^) where fraction cleaved = (sum of cleavage product intensities)/(sum of uncleaved and cleaved product intensities).

### Polysome profiling

HEK cells cultured in 10 cm plates were washed with 10 mL DPBS before lysis with 100‐400 μL ice cold polysome buffer (20 mm Tris pH 7.4, 150 mm NaCl, 5 mm MgCl_2_, 1 mm DTT, and 100 μg·mL^−1^ cycloheximide) with 1% Triton X‐100 and 25 U·mL^−1^ TURBO DNase (Thermo Fisher Scientific). Cells were scraped off plates in lysis buffer and incubated on ice in microcentrifuge tubes for 10 min. Lysates were spun at 10 min at 20 000 ***g***, and the supernatants were normalized using the Quant‐iT RiboGreen RNA Assay Kit (Thermo Fisher Scientific) to concentrations between 50 and 250 ng·μL^−1^.

The 6 mL 50% (w/v) sucrose in polysome buffer was layered under 6 mL 10% sucrose solution in polysome buffer in 14 × 89 mm ultracentrifuge tubes (VWR), and 10–50% sucrose gradients were created using a Gradient Master (BioComp Instruments, Fredericton, NB, Canada) with rotation set at 81.5°, speed 16 for 1 : 58. Two hundred microliter normalized cell lysate was layered on top of the gradients, and the gradients were loaded into Sw41 Ti rotor buckets (Beckman Coulter, Brea, CA, USA) and spun at 36 000 r.p.m. (~ 250 000 ***g***) for 2.5 or 3 h at 4 °C in a L8‐M Ultracentrifuge (Beckman). Sucrose gradients were pumped through the Gradient Master at 0.2 mm·s^−1^, and UV absorbance at 254 nm was measured using a BioRad EM‐1 Econo UV Monitor connected to a laptop running the logger lite software package (Vernier, Beaverton, OR, USA). Proteins were extracted for western blots using the methanol/chloroform protocol detailed in the Click‐it Metabolic Labeling Reagents for Proteins manual (Invitrogen), and pellets were boiled at 95 °C in 1× Laemmli buffer before SDS/PAGE.

### RT‐qPCR

RNA was extracted from cells using the Direct‐zol™ RNA MiniPrep Kit (Zymo, Irvine, CA, USA) according to the manufacturer's instructions. One microgram total RNA was used for reverse transcription with Superscript III First Strand Synthesis SuperMix (Thermo Fisher Scientific). qRT‐PCR was performed using Fast SYBR Green Master Mix (Thermo Fisher Scientific) on a StepOnePlus Real‐Time PCR System (Thermo Fisher Scientific). *C*_t_ values from target genes were normalized to *GAPDH*, and the expression of each gene was represented as $2^{‐\Delta \Delta C_{t}}$ relative to the reference sample. The *JAK2* qPCR primers were AACTGCATGAAACAGAAGTTCTT (forward) and GCATGGCCCATGCCAACTGT (reverse), the *GAPDH* qPCR primers were TGCACCACCAACTGCTTAG (forward) and GGATGCAGGGATGATGTTC (reverse), and *RPS27A* qPCR primers were TGTCTCTTCCTTTTCCTCAACC (forward) and CTATCGTATCCGAGGGTTCAA (reverse).

### Bulk translation assays

Six well or 10 cm plates of HEK cells were washed with PBS then placed in 25 μm Click‐IT l‐azidohomoalanine (Thermo Fisher Scientific) in DMEM, high glucose, no glutamine, no methionine, and no cysteine (Thermo Fisher Science) with 10% FBS for 2 h. Cells were trypsinized then pelleted at 400 ***g*** for 5 min. Cells were washed three times with PBS before being lysed in 100 or 200 μL lysis buffer (1% SDS, 50 mm Tris HCl, pH 8.0, 1× Halt Protease Inhibitor Cocktail; Thermo Scientific) with 150 U·mL^−1^ benzonase nuclease to digest DNA and RNA or 25 U·mL^−1^ TURBO DNase (Thermo Fisher Scientific). Lysates were incubated for 30 min on ice, vortexed for 5 s, and spun at 18 000 ***g*** for 10 min at 4 °C. Protein content of the supernatants was normalized using the Pierce BCA Protein Assay (Thermo Fisher Scientific). One microliter 10 mm IRDye 800CW‐DBCO was added to the lysates, and the lysates were incubated for 2 h at RT. Unbound IR Dye was removed using a Zeba Column, 7K MWCO, 0.5 mL (Thermo Fisher Scientific), and lysates were boiled in 1× LDS sample buffer and run on 4–20% Tris‐Glycine gels as detailed for the third SDS/PAGE method in the 'western blotting' section. For dot blot analysis, a Bio‐Dot Microfiltration Apparatus (Bio‐Rad) was used according to the manufacturer's protocol with 20 μL sample added to wells. Membranes and SDS/PAGE gels were imaged on a LI‐COR Odyssey CLx Imager.

### Ribosome profiling and RNA‐seq

Paired ribosome profiling and RNA‐seq experiments were conducted on HEK 293 cells lysed 36 and 72 h after Cas9 or dCas9 RNP electroporation. Cas9 and dCas9 complexed with sgIntron, a guide targeting intron 12 of *JAK2*, were electroporated using the protocols detailed in ‘Cas9 RNP Electroporations’. Four small‐scale electroporations were pooled directly into one 10 cm plate to create one biological replicate with each experimental condition having two biological replicates. Due to recent reports about IVT guide RNAs inducing interferon responses in cells [31, 32], synthetic sgRNAs (Synthego) were used at the 36 h time point.

Ribosome profiling was conducted as detailed in Ref. [33] with the following modifications. Since Epicentre discontinued the yeast 5′‐deadenylase (Cat# DA11101K) used in Ref. [33], we cloned a 5′‐deadenylase (*HNT3*) from the thermotolerant yeast *Kluyveromyces marxianus* into the pET His6 TEV LIC cloning vector (2B‐T) backbone (gift from Scott Gradia to Addgene). Recombinant 6xHis‐TEV‐Km‐HNT3 was purified from *Escherichia coli* using a nickel column (HisTrap FF Crude column; GE Life Sciences, Marlborough, MA, USA). Protein eluted from the column with imidazole was cleaved with TEV protease, and the residual His tag was removed using a nickel column. The recombinant protein subsequently purified using size exclusion chromatography (Sephacryl S‐300 16/60 column; GE Life Sciences). 0.5 μL of purified protein was added in place of the yeast 5′‐deadenylase during ribosome profiling, and the reaction was incubated at 37 °C instead of 30 °C. We also deviated from Ref. [33] by using CircLigase I (Lucigen) instead of CircLigase II (Lucigen). We made this change after concerns about the nucleotide bias of CircLigase II were reported in Ref. [34]. Therefore, we reverted to using CircLigase I as previously detailed in Ref. [35] with a 2 h incubation step.

Total RNA for mRNA‐seq was isolated from 50 μL cell lysate using the DirectZol™ RNA MiniPrep Kit (Zymo) according to the manufacturer's protocol. Sequencing libraries were prepared using the TruSeq Stranded Total RNA Library Kit with Ribo‐Zero Gold (Illumina, San Diego, CA, USA). Ribosome profiling and RNA‐seq libraries were sequenced as 50 nt single‐end reads on an Illumina HiSeq 4000.

Reads from ribosome profiling were processed as detailed in Ref. [33]. Ribosome profiling and RNA‐seq reads from the 36 h time point were aligned with hisat2 [36] to the Human GENCODE Gene Release GRCh38.p2 (release 22); reads from the 72 h time point were aligned with tophat [37] to GRCH38.p7 (release 25). Alignments were indexed using samtools [38], and the number of reads per transcript was tabulated using fp‐count [39] with the basic gene annotations from GRC38.p2 (36 h) and GRCh38.p7 (72 h). Differential changes in gene expression were calculated using deseq2 [40] with a cutoff of FDR < 0.1 for per‐gene significance. Translational efficiency (the ratio of ribosome footprints to mRNA‐seq transcripts) calculations and significance tests were made in deseq2 using a design matrix that tested the ratio of ratios (design = ~ A + B + A:B, where A was Cas9 type and B was library type) with FDR < 0.1.

Cumulative distribution functions and Mann–Whitney–Wilcoxon tests with ribosome profiling and RNA‐seq data were calculated in rstudio (RStudio, Boston, MA, USA). Three gene lists were used for this analysis: ISR targets, ribosome proteins, and DSB break repair genes. ISR targets are the 78 genes identified by Ref. [20] to have a statistically significant, greater than twofold change in translational efficiency after tunicamycin treatment. (6 of the 78 genes were removed from analysis because we were unable to identify corresponding GRCh38 Ensembl gene IDs from the original GRCh37 UCSC gene IDs listed in [20].) DSB break repair genes are the union of genes annotated as DSB repair genes in [41] and those listed on the University of Pittsburgh Cancer Institute's DNA Repair Database website (https://dnapittcrew.upmc.com/db/index.php).

### Generating RPS27A‐SBP Flp‐In cell lines

RNA from HEK cells was isolated using the DirectZol RNA MiniPrep Kit (Zymo) according to the manufacturer's protocol. cDNA was generated using SuperScript II Reverse Transcriptase (Thermo Fisher Scientific), and coding regions of RPS27A without the N‐terminal ubiquitin sequence was PCR amplified and cloned into a pcDNA5/FRT/TO vector backbone (Invitrogen) that had been previously modified to have a constitutive CMV promoter and C‐terminal SBP‐tag.

To generate stable transgenic cell lines, 1 × 10^6^ HEK Flp‐In T‐Rex Cells (Invitrogen) were electroporated using a Lonza 4D Nucleofector in according to the Amaxa 4D‐Nucleofector™ Protocol for HEK293 (Lonza) for large cuvettes with 1.8 μg pOG44 Flp‐Recombinase Expression Vector (Invitrogen) and 0.2 μg pCMV‐RPS27A‐SBP. Two days after electroporation, cells were passaged and placed on media containing 5 μg·mL^−1^ blasticidin (Invitrogen) and 10 μg·mL^−1^ Hygromycin B (Thermo Fisher Scientific) until all cells from a control plate electroporated with pmaxGFP™ Vector (Lonza) were dead. Flp‐In cell lines were validated using anti‐SBP westerns and Sanger sequencing of the transgenic insert.

### Quantification and statistical analysis

Bar graphs, scatterplots, and cumulative distribution function plots were created with rstudio version 1.0.136 running r version 3.3.2 or prism v9 (GraphPad, San Diego, CA, USA). Standard statistical analyses such as standard deviation calculations and Mann–Whitney–Wilcoxon tests were conducted in r, and one‐way ANOVAs for qPCR data were calculated using prism. FDR values for RNA‐seq and ribosome profiling were calculated using the Wald test in deseq2 as described in Ref. [40]. Statistical details of experiments such as sample size (*n*) can be found in the figures and figure legends. For this article, *n* is the number of biological replicates and SD is the standard deviation assuming a normal distribution.

## Conflict of interest

Jacob E. Corn is a co‐founder of Spotlight Therapeutics.

## Author contributions

CR wrote and edited manuscript; designed figures; performed western blotting, polysome profiling, ribosome profiling, bulk tranlsation, and RNA‐seq experiments; and analyzed ribosome profiling and RNA‐seq datasets. EZ conducted western blotting, qPCR, bulk translation, and genome‐editing efficiency experiments; edited manuscript. PAF constructed RPS27‐SBP Flp‐In cell lines; and edited manuscript. ZAM performed molecular biology for the 36‐h RNA‐seq experiment. SGF performed molecular biology for the Cas9 eIF2ɑ phosphorylation experiments. NTI analyzed ribosome profiling and RNA‐seq datasets; supervised CR, SGF, and ZAM; and edited manuscript. JEC wrote and edited manuscript and supervised EZ.

## Supporting information


**Fig. S1.** Genome editing initiates a translational response that precedes changes in transcript abundance.
**Table S1.** Ribosome Profiling and RNA‐seq DESeq2 Analysis (related to Figure 4). Sheet 1: Ribosome Profiling, 36 Hours; Sheet 2: RNA‐seq, 36 Hours; Sheet 3: Translational Efficiency, 36 Hours; Sheet 4: Ribosome Profiling, 72 Hours; Sheet 5: RNA‐seq, 72 Hours; Sheet 6: Translational Efficiency, 72 Hours.
**Table S2.** Target Gene Lists for CDF Plots (related to Figure 4). Sheet 1: Integrated Stress Response (ISR) Genes[20]. Sheet 2: Ribosome Protein Genes. Sheet 3: DSB Repair Genes, union of genes annotated as DSB repair genes from [41] and University of Pittsburgh Cancer Institute's DNA Repair Database.

## Data Availability

Ribosome profiling and mRNA‐Seq data are available from NCBI GEO, with Accession #GSE122615.
