# Supplementary material for: Double stranded DNA breaks and genome editing trigger loss of ribosomal protein RPS27A
Source: FEBS J. 2022 Jan 10;289(11):3101–14. doi: 10.1111/febs.16321 (PMC9295824; doi:10.1111/febs.16321)
Supplement: Supplementary file 1 — Fig. S1. Genome editing initiates a translational response that precedes changes in transcript abundance. Table S1. Ribosome Profiling and RNA‐seq DESeq2 Analysis (related to Figure 4). Sheet 1: Ribosome Profiling, 36 Hours; Sheet 2: RNA‐seq, 36 Hours; Sheet 3: Translational Efficiency, 36 Hours; Sheet 4: Ribosome Profiling, 72 Hours; Sheet 5: RNA‐seq, 72 Hours; Sheet 6: Translational Efficiency, 72 Hours. Table S2. Target Gene Lists for CDF Plots (related to Figure 4). Sheet 1: Integrated Stress Response (ISR) Genes[20]. Sheet 2: Ribosome Protein Genes. Sheet 3: DSB Repair Genes, union of genes annotated as DSB repair genes from [41] and University of Pittsburgh Cancer Institute's DNA Repair Database. [file FEBS-289-3101-s001.zip › febs16321-sup-0001-FigS1.pdf]

FEBS PRESS
science publishing by scientists
WILEY
The FEBS Journal
www.febsjournal.org

Double stranded DNA breaks and genome editing trigger loss of ribosomal protein RPS27A

Celeste Riepe, Elena Zelin, Phillip A. Frankino, Zuriah A. Meacham, Samantha G. Fernandez, Nicholas T. Ingolia and Jacob E. Corn

DOI: 10.1111/febs.16321

Figure S1. Genome editing initiates a translational response that precedes changes in transcript abundance

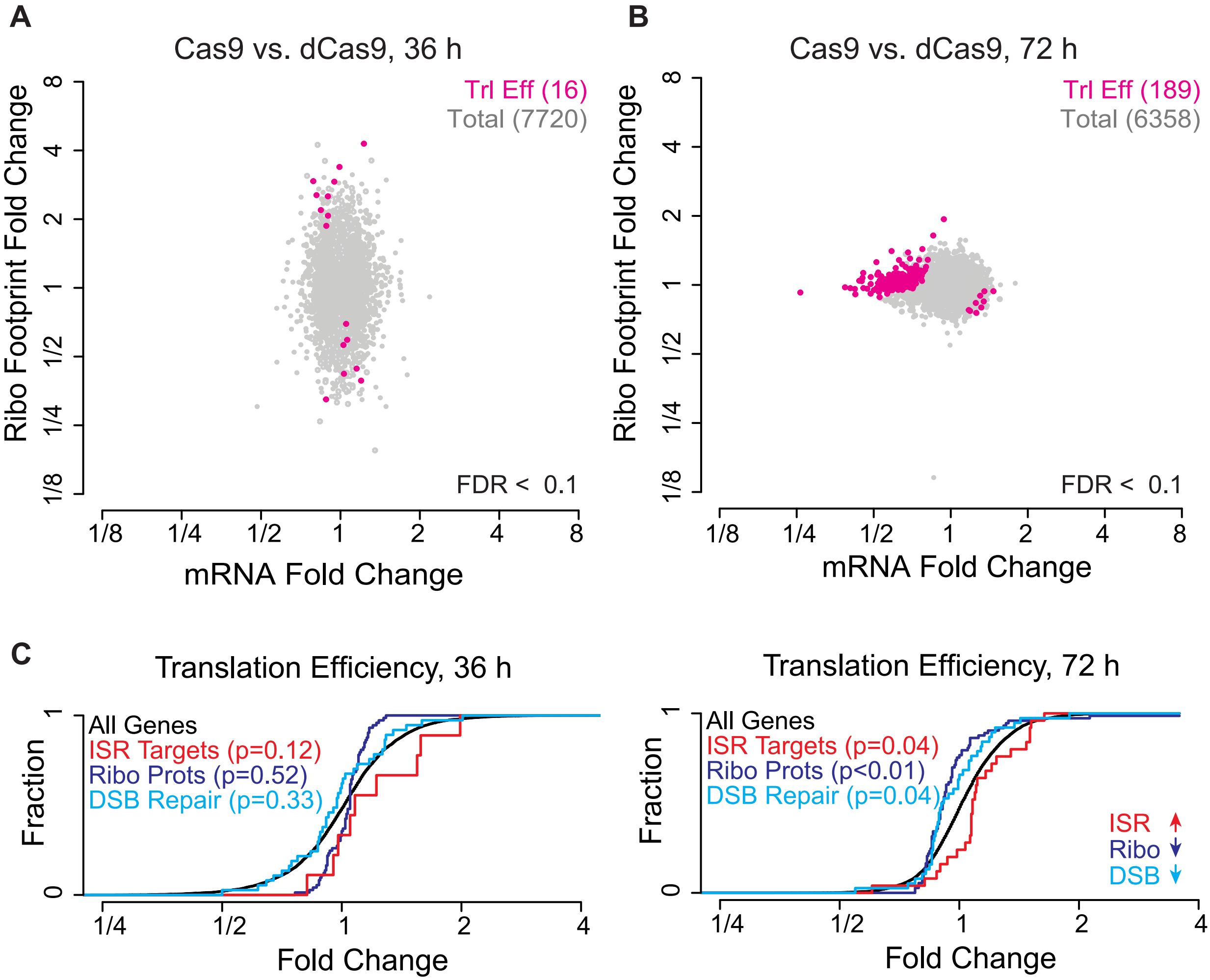

(A) Changes in translational efficiency, the ratio of ribosome footprints to mRNA transcripts at 36 hours after Cas9-sgIntron electroporation. Pink = genes with significant changes in translational efficiency (Wald test, FDR < 0.1).

(B) As in (A) for 72-hour ribosome profiling and RNA sequencing data.

(C) Cumulative distribution function (CDF) plots of translational efficiency for ribosomal protein genes (Ribo), integrated stress response targets (ISR), and DSB repair genes after Cas9-sgIntron electroporation. p-values were calculated using the Mann-Whitney-Wilcoxon rank sum test. See Table S2 for target set gene lists.
